# *Helicobacter pylori* infection, serum pepsinogens as markers of atrophic gastritis, and leukocyte telomere length: a population-based study

**DOI:** 10.1186/s40246-019-0217-3

**Published:** 2019-07-22

**Authors:** Khitam Muhsen, Ronit Sinnreich, Dafna Merom, Hisham Nassar, Dani Cohen, Jeremy D. Kark

**Affiliations:** 1 Department of Epidemiology and Preventive Medicine, School of Public Health, Sackler Faculty of Medicine, Tel Aviv University Ramat Aviv, Ramat Aviv, 6139001 Tel Aviv, Israel; 2 Hadassah School of Public Health and Community Medicine, Hebrew University, Jerusalem, Israel; 3 Western Sydney University, Sydney, Australia; 4 St. Joseph Hospital, East Jerusalem and Department of Cardiology, Hadassah-Hebrew University Medical Center, Ein Kerem, Jerusalem, Israel

**Keywords:** Leukocyte telomere length, *Helicobacter pylori*, Serum pepsinogens, Atrophic gastritis, Cytotoxin-associated gene A antigen

## Abstract

**Background:**

Persistent infections that induce prolonged inflammation might negatively affect the leukocyte telomere length (LTL); however, the role in LTL of *Helicobacter pylori* (*H. pylori*) infection, which persistently colonizes the stomach, remains unknown.

The study objective was to examine associations of sero-prevalence of *H. pylori* immunoglobulin G (IgG) antibody and serum pepsinogens (PGs), as markers of atrophic gastritis, with LTL.

A cross-sectional study was performed among 934 Arab residents of East Jerusalem, aged 27–78 years, randomly selected from Israel’s national population registry. Sera were tested for *H. pylori* IgG and PG levels by ELISA. LTL was measured by southern blots. Multiple linear regression models were fitted to adjust for sociodemographic and lifestyle factors.

**Results:**

LTL decreased significantly with age (*p* < 0.001) and was shorter in men than women (*p* = 0.032). The mean LTL was longer in *H. pylori* sero-positive persons than negative ones: mean difference 0.13 kb (95% CI 0.02, 0.24), *p* = 0.016. Participants with atrophic gastritis (PGI < 30 μg/L or a PGI: PGII < 3.0) had shorter LTL than did those without: mean difference − 0.18 (95% CI − 0.32, − 0.04). The difference was of larger magnitude between persons who had past *H. pylori* infection (sero-negative to *H. pylori* IgG antibody) and atrophic gastritis, compared to those who were *H. pylori* sero-negative and did not have atrophic gastritis: mean difference − 0.32 kb (95% CI − 0.55, − 0.10). This association remained significant after adjustment for age, sex, and religiosity: beta coefficient − 0.21 kb (95% CI − 0.41, − 0.001), *p* = 0.049. The results were similar after further adjustment for lifestyle factors. In bivariate analysis, mean LTL was longer in physically active persons than non-active ones, and shorter in persons with than without obesity; however, these differences were diminished and were not significant in the multivariable model.

**Conclusions:**

*H. pylori* IgG sero-positivity per se was not related to reduced LTL. However, persons with past *H. pylori* infection (i.e., lacking *H. pylori* IgG serum antibody) and with serological evidence of atrophic gastritis, had a significantly shorter LTL than did those without atrophic gastritis.

**Electronic supplementary material:**

The online version of this article (10.1186/s40246-019-0217-3) contains supplementary material, which is available to authorized users.

## Background

Telomeres are repetitive nucleotides at the ends of eukaryotic chromosomes [1, 2] that are essential for genomic stability. Telomere length shortens with every cell division, due to the inability of DNA polymerase to extend DNA ends [1, 3]. Telomeres that shorten beyond a critical level cause cellular senescence [1, 3]; thus, telomere length has a considerable impact on human health [4].

Leukocyte telomere length (LTL) is commonly assessed in epidemiological studies [4]. LTL is affected by heredity [5, 6]; decreases with age [5, 7, 8]; is longer in females than males [7, 8]; correlates positively with paternal age at conception [6]; and varies by race, being longer, for example, in persons of African ancestry than individuals of European ancestry [4, 7, 9].

Shorter LTL was consistently linked to increased risk of atherosclerosis [10–15] and reduced survival [16–18]. The association between LTL and cancer risk is less consistent [19]. Some studies showed an increased risk for cancer in relation to short LTL [20, 21], while in other studies longer LTL was associated with increased cancer risk [22]. Given the importance of telomere length in human health, searching for modifiable determinants of LTL is highly desirable. Persons of low socioeconomic status displayed shorter LTL than did persons of high socioeconomic status [23]. Smokers were found to have shorter LTL than never or former smokers [24, 25]. Obesity [25] and higher body mass index (BMI) [26] were associated with shorter telomeres [26], while a positive association was found of physical activity with telomere length [27, 28]. An inverse association was shown between dietary caloric intake and LTL in men [29]. Persistent infections have been suggested to play a role in telomere length through the induction of oxidative stress and inflammation [30, 31]. For example, associations were reported of exposure to cytomegalovirus (CMV), herpes simplex virus type 1 (HSV-1), and human herpes virus 6 with greater LTL attrition in healthy adults [31], although these associations were not affected by systemic non-specific inflammatory markers such as C-reactive protein and interleukin 6 [31]. If infectious agents are truly associated with LTL, associations would be expected between tissue damage induced by these pathogens and LTL. *Helicobacter pylori* (*H. pylori*), a gram-negative bacterium that colonizes the stomach, can serve as a model to examine this hypothesis. *H. pylori* infection is acquired in childhood [32]. While *H. pylori* cause chronic gastritis in almost all infected persons, some present with peptic ulcers and gastric cancer in adulthood, especially those infected with strains that express cytotoxin-associated gene A (CagA) virulence antigen (reviewed in [33, 34]). Pepsinogen I (PGI) and PGII, pro-enzymes of pepsin, are secreted into the gastric lumen, and small amounts can be found in the serum [35, 36]. With increasing severity of *H. pylori* gastritis, serum PGI and PGII levels increase, but when atrophic gastritis ensues, the PGI level and the PGI:PGII ratio decrease. Serum PGs can predict atrophic gastritis and gastric cancer [37–41]. The objective of the current study was to examine associations of the sero-prevalence of *H. pylori* immunoglobulin G (IgG) antibody and serological evidence of atrophic gastritis with LTL in a general population sample.

## Results

### Description of the study sample

Overall, 934 participants (53.1% men) were tested for both *H. pylori* IgG antibody and LTL. The age at examination ranged from 27.0 to 78.0 years, with a mean of 52.0 years (standard deviation [SD] 13.9), which was similar among men and women. Most (64.2%) participants had not completed high school; this was more true for women (70.3%) than men (58.8%). Most participants (62.4%) defined themselves as traditional/secular: 67.5% and 56.6% in men and women, respectively. Overall, 82.0% of the participants reported being married; 94.2% and 68.1% among men and women, respectively. The overall prevalence of smoking was 26.0%: 40.8% in men and 9.0% in women; the respective figures for obesity were 44.1%, 31.9%, and 58.0% (Table 1).

**Table 1:** Characteristics of the study sample

	Overall, *n* (%)	Men, *n* (%)	Women, *n* (%)
Total	934	496	438
Mean age, years, (SD)	52.0 (13.9)	51.9 (14.0)	52.0 (13.8)
Age groups, years			
27–34	127 (13.6)	71 (14.3)	56 (12.8)
35–44	191 (20.4)	97 (19.6)	94 (21.5)
45–54	205 (21.9)	110 (22.2)	95 (21.7)
55–64	195 (20.9)	100 (20.2)	95 (21.7)
65–78	216 (23.1)	118 (23.8)	98 (22.3)
Education			
Did not complete high school	598 (64.2)	291 (58.8)	307 (70.2)
Completed high school	210 (22.5)	118 (23.8)	92 (21.1)
Academic education	124 (13.3)	86 (17.4)	38 (8.7)
Number of siblings			
0–3	87 (9.3)	45 (9.1)	42 (9.6)
4–7	423 (45.3)	222 (44.8)	201 (45.9)
≥ 8	423 (45.3)	228 (46.1)	195 (44.5)
Religiosity			
Religious	350 (37.6)	161 (32.5)	189 (43.4)
Traditional/secular	580 (62.4)	334 (67.5)	246 (56.6)
Marital status*			
Married	764 (82.0)	467 (94.2)	297 (68.1)
Not married	168 (18.0)	29 (5.8)	139 (31.9)
Smoking			
≥ 1 cigarettes/day	241 (26.0)	202 (40.8)	39 (9.0)
No smoking/other	687 (74.0)	293 (59.2)	394 (91.0)
Obesity			
BMI < 30 kg/m^2^	522 (55.9)	338 (68.1)	184 (42.0)
BMI ≥ 30 kg/m^2^	412 (44.1)	158 (31.9)	254 (58.0)
Sufficient physical activity level			
No	180 (19.3)	68 (13.7)	112 (25.6)
Yes	754 (80.7)	428 (86.3)	326 (74.4)
High physical activity level			
No	265 (28.4)	111 (22.4)	154 (35.2)
Yes	669 (71.6)	385 (77.6)	284 (64.8)

*BMI* body mass index; *SD* standard deviation

*Not married included persons who defined themselves as single, divorced, or widowed

### Mean LTL according to demographic and lifestyle factors

The LTL values ranged from 4.72 kb to 8.53 kb, with a mean of 6.76 kb (SD 0.61). The mean LTL was longer in women than men (*p* = 0.073). The mean LTL was longest in the youngest age group (27–34 years), 7.18 kb (SD 0.55), and decreased progressively in older age groups, reaching 6.40 kb (SD 0.55) at age 65–78 years (*p* < 0.001). All pairwise comparisons between the age groups were statistically significant by the Bonferroni test. The mean LTL differed significantly (*p* = 0.007) according to education, with a mean 6.72 kb (SD 0.58) among participants who had not completed high school, 6.83 kb (SD 0.61) in those who had completed high school (*p* = 0.055 by the Bonferroni test), and 6.87 kb (SD 0.71) in participants with academic education (*p* = 0.033 by the Bonferroni test). Persons who defined themselves as religious had shorter mean LTL than persons who defined themselves as traditional or secular (*p* < 0.001). Married individuals had longer mean LTL than unmarried ones: mean difference 0.11 (95% confidence intervals [CI] 0.01, 0.21), *p* = 0.039. Persons with obesity had shorter mean LTL, 6.71 kb (SD 0.60), than persons without obesity, 6.80 kb (SD 0.62), (*p* = 0.025). Physically active persons had longer mean LTL than non-active ones (*p* < 0.001); this association was found both for physical activity that was defined as sufficient and as high level. Mean LTL was not found to differ significantly according to the number of siblings (*p* = 0.2) and smoking (*p* = 0.6) (Table 2). Differences in the expected directions in mean LTL, according to age, religiosity, obesity, and high level of physical activity were observed in both men and women. In women, mean LTL differed significantly (*p* = 0.001) according to educational level, being the longest among those with academic education; but such difference was not significant (*p* = 0.3) in men (*p* for interaction 0.2). The difference in mean LTL according to marital status was significant in women only (*p* for interaction 0.009). The difference in mean LTL according to a modest level of physical activity level was significant among women only (*p* for interaction 0.07). No significant interactions were found between the other independent variables and sex (see Additional file 1).

**Table 2:** Mean leukocyte telomere length (kb) according to sociodemographic and lifestyle factors

	Total	Mean (SD)	Mean difference (95% CI)	*p* value
Sex				0.073
Men	496	6.73 (0.63)	− 0.07 (− 0.15, 0.01)	
Women	438	6.80 (0.59)	Reference	
Age, years	df = 4			< 0.001^*^
27–34	127	7.18 (0.55)	Reference	
35–44	191	7.00 (0.54)	− 0.18 (− 0.36, − 0.01)	
45–54	205	6.80 (0.55)	− 0.39 (− 0.56, − 0.21)	
55–64	195	6.62 (0.56)	− 0.57 (− 0.75, − 0.39)	
65–78	216	6.40 (0.55)	− 0.78 (− 0.96, − 0.61)	
Education	df = 2			0.007^**^
Did not complete high school or less	598	6.72 (0.58)	Reference	
Completed high school	210	6.83 (0.61)	0.11 (− 0.002, 0.23)	
Academic education	124	6.87 (0.71)	0.15 (0.01, 0.30)	
Number of siblings	df=2			0.2^***^
0–3	87	6.67 (0.60)	Reference	
4–7	423	6.75 (0.63)	0.08 (− 0.09, 0.25)	
≥ 8	423	6.79 (0.59)	0.12 (− 0.05, 0.30)	
Religiosity				< 0.001
Religious	350	6.67 (0.61)	− 0.15 (− 0.23, − 0.07)	
Traditional/secular	580	6.82 (0.60)	Reference	
Marital status^****^				0.039
Married	764	6.78 (0.62)	0.11 (0.01, 0.21)	
Not married	168	6.67 (0.56)	Reference	
Smoking				0.6
≥ 1 cigarettes/day	241	6.78 (0.59)	0.02 (− 0.07, 0.11)	
No smoking/other	687	6.76 (0.62)	Reference	
Obesity				0.025
BMI < 30 kg/m^2^	522	6.80 (0.62)	0.09 (0.01, 0.17)	
BMI ≥ 30 kg/m^2^	412	6.71 (0.60)	Reference	
Sufficient physical activity level				
No	180	6.60 (0.59)	− 0.20 (− 0.29, − 0.10)	< 0.001
Yes	754	6.80 (0.61)	Reference	
High physical activity level				< 0.001
No	265	6.62 (0.61)	− 0.20 (− 0.30, − 0.10)	
Yes	669	6.82 (0.60)	Reference	

*BMI* body mass index; *CI* confidence intervals; *df* degrees of freedom

^*^ANOVA for the difference between the groups. Bonferroni test 27–34 vs. 35–44 (*p* = 0.035), 45–54 vs. 55–64 (*p* = 0.013), *p* < 0.01 for all other pairwise comparisons between the age groups

^**^ANOVA for the difference between the groups. Bonferroni test: did not complete high school vs. completed high school (*p* = 0.055). Did not complete high school vs. academic degree (*p* = 0.033). Completed high school vs. academic education (*p* = 1.0)

^***^ANOVA for the difference between the groups. Bonferroni test *p* > 0.2 for all pairwise

^****^Not married included persons who reported being single, widowed, or divorced

### *H. pylori* sero-status, atrophic gastritis, and LTL

*H. pylori* IgG sero-positivity was found in 780/934 (83.4%) participants and atrophic gastritis in 81/927 (8.7%). In persons with a past *H. pylori* infection (lacked *H. pylori* IgG serum antibody), atrophic gastritis was found in 37 (4.0%), atrophic gastritis and *H. pylori* sero-positivity was evident in 44 (4.7%), and *H. pylori* sero-positivity without serological evidence of atrophic gastritis in 731 (78.9%), while 115 (12.4%) tested negative for *H. pylori* and lacked serological evidence for atrophic gastritis

The mean LTL was longer in *H. pylori* IgG sero-positive persons than in sero-negative ones: 6.78 kb (SD 0.59) vs. 6.65 kb (SD 0.66), *p* = 0.016; the difference was significant only when comparing *H. pylori* sero-positive persons who had CagA IgG serum antibody with those who were sero-negative (*p* = 0.018 by Bonferroni test). Participants who had serological evidence of atrophic gastritis had significantly shorter LTL (6.60 kb [SD 0.65]) than did participants without atrophic gastritis: 6.78 kb (SD 0.60) (*p* = 0.011). The shortest LTL was found in participants who had past *H. pylori* infection (sero-negative to *H. pylori* IgG antibody) and atrophic gastritis (6.40 kb [SD 0.67]); this compared to persons who were *H. pylori* sero-negative without atrophic gastritis (6.72 kb [SD 0.63]), those who were *H. pylori* sero-positive without atrophic gastritis (6.79 kb [SD 0.60]), and those who were *H. pylori* sero-positive with atrophic gastritis (*p* = 0.028, *p* = 0.001, and *p* = 0.037, respectively, by the Bonferroni test) (Table 3).

**Table 3:** Mean leukocyte telomere length (kb) according to *H. pylori* sero-status and serological evidence of atrophic gastritis

	Total	Mean (SD)	Mean difference (95% CI)	*p* value
*H. pylori* IgG sero-status				0.016^*^
*H. pylori* negative	154	6.65 (0.66)	Reference	
*H. pylori* positive	780	6.78 (0.60)	0.13 (0.02, 0.24)	
*H. pylori/*CagA IgG sero-status	df = 2			0.023^**^
*H. pylori* negative	154	6.65 (0.66)	Reference	
*H. pylori* positive CagA negative	452	6.76 (0.60)	0.1 (− 0.01, 0.22)	0.19^***^
*H. pylori* positive CagA positive	328	6.82 (0.59)	0.16 (0.05, 0.28)	0.018^***^
Atrophic gastritis (PGI < 30 μg/L or PGI:PGII < 3.0)^§^				0.011^*^
No	846	6.78 (0.60)	− 0.18 (− 0.32, − 0.04)	
Yes	81	6.60 (0.65)	Reference	
*H. pylori* sero-status/atrophic gastritis^**** §^	df = 3			0.002^**^
*H. pylori* negative no atrophic gastritis	115	6.72 (0.63)	Reference	
*H. pylori* positive no atrophic gastritis	731	6.79 (0.60)	0.07 (− 0.05, 0.18)	1.0^****^
*H. pylori* positive plus atrophic gastritis	44	6.77 (0.58)	0.05 (− 0.16, 0.26)	1.0^****^
Past *H. pylori* infection (IgG sero-negatives) plus atrophic gastritis	37	6.40 (0.67)	− 0.32 (− 0.55, − 0.10)	0.028^****^

*CagA* cytotoxin-associated gene A; *CI* confidence intervals; *df* degrees of freedom; *IgG* immunoglobulin G; *PG* pepsinogen; *SD* standard deviation

^*^p value by Student’s *t* test

^**^ANOVA for the difference between the groups

^***^Bonferroni for multiple comparisons correction compared to *H. pylori*-negative participants

^****^Bonferroni for multiple comparisons correction compared to participants who were *H. pylori* negative and no atrophic gastritis. *H. pylori* positive no atrophic gastritis vs. past *H. pylori* infection plus atrophic gastritis (*p* = 0.001). *H. pylori* positive plus atrophic gastritis vs. past *H. pylori* infection (IgG sero-negatives) plus atrophic gastritis (*p* = 0.037)

^**§**^Information on atrophic gastritis was missing for 7 participants

A multiple linear regression model that included the variable *H. pylori* sero-status/atrophic gastritis and adjusted for age, sex, and religiosity was statistically significant (*F* statistic = 26.24, *p* < 0.001) with an adjusted *R*^2^ of 0.198 (Table 4). This model showed an inverse dose-response relationship between age and LTL, and significantly shorter LTL in men than in women; beta coefficient − 0.08 kb (95% CI − 0.15, − 0.01), *p* = 0.032. Compared to participants who were *H. pylori* sero-negative without atrophic gastritis, those with past *H. pylori* infection (sero-negative to *H. pylori* IgG antibody) and with atrophic gastritis had shorter LTL: beta coefficient − 0.21 kb (95% CI − 0.41, − 0.001), *p* = 0.049; the difference was not significant in *H. pylori* sero-positive persons either with (*p* = 0.10) or without atrophic gastritis (*p* = 0.3). The mean LTL was shorter in religious than traditional/secular participants; beta coefficient − 0.07 kb (95% CI − 0.14, 0.01), *p* = 0.078 (Table 4). The values of variance inflation factor (VIF) in this model ranged from 1 to 2, suggesting no collinearity.

**Table 4:** Multiple linear regression model of adjusted associations of demographic factors, *H. pylori* sero-status, and serological evidence of atrophic gastritis with leukocyte telomere length (kb)

	Beta coefficient (95% CI)	*p* value
Sex		
Males	− 0.08 (− 0.15, − 0.01)	0.032
Females	Reference	
Age, years		
27–34	Reference	
35–44	− 0.19 (− 0.31, − 0.06)	0.003
45–54	− 0.39 (− 0.51, − 0.26)	< 0.001
55–64	− 0.56 (− 0.69, − 0.44)	< 0.001
65–78	− 0.76 (− 0.89, − 0.64)	< 0.001
Religiosity		
Religious	− 0.07 (− 0.14, 0.01)	0.078
Traditional/secular	Reference	
*H. pylori* sero-status/atrophic gastritis^*^		
*H. pylori* negative no atrophic gastritis	Reference	
*H. pylori* positive no atrophic gastritis	0.05 (− 0.06, 0.16)	0.3
*H. pylori* positive plus atrophic gastritis	0.16 (− 0.03, 0.35)	0.10
Past *H. pylori* infection (IgG sero-negatives) plus atrophic gastritis	− 0.21 (− 0.41, − 0.001)	0.049

*CI* confidence intervals; *IgG* immunoglobulin G

^*^Atrophic gastritis was defined as serum pepsinogen (PG) *I* < 30 μg/L or PGI:PGII < 3.0

Model summary: adjusted *R*^2^ = 0.198, degrees of freedom = 9, (*F* statistic = 26.24), *p* < 0.001

Adjusted for the variables in the table

An additional model that included the variable *H. pylori*/atrophic gastritis sero-status and the following covariates; age, sex, religiosity, education, marital status, number of siblings, smoking, obesity, and physical activity showed similar results with regard to the associations of age, sex, and *H. pylori*/atrophic gastritis sero-status with LTL (see Additional file 2). However, no significant differences in LTL were observed according to obesity (*p* = 0.6), smoking (*p* = 0.7), physical activity (*p* = 0.5), marital status (*p* = 0.8), number of siblings, or education. The adjusted *R*^2^ of this model was 0.193, (*F* statistic = 13.86, *p* < 0.001). The VIF values ranged from 1 to 2, suggesting no collinearity. No significant interactions were found between *H. pylori*-atrophic gastritis sero-status with sex (*p* = 0.2), age (*p* = 0.12), education (*p* = 0.11), obesity (*p* = 0.2), physical activity (*p* = 0.12), and smoking (*p* = 0.8). Therefore, the interaction terms were excluded from the final model.

## Discussion

We examined associations of *H. pylori* IgG antibody sero-positivity and serological evidence of atrophic gastritis with LTL in a general Arab population sample while assessing the role of sociodemographic and lifestyle factors.

As expected, a significantly shorter LTL was observed in older vs. younger participants and in men vs. women, thus confirming previous findings [5, 7, 8]. Exposure to *H. pylori* infection per se was not associated with shorter LTL. On the contrary, *H. pylori* IgG sero-positivity, especially CagA phenotype, was associated with longer LTL. Serological evidence of atrophic gastritis was associated with shorter LTL, the difference was driven by participants who tested negative to *H. pylori* but had atrophic gastritis. The longer LTL in *H. pylori* sero-positive participants can be explained by the higher prevalence of serological evidence of atrophic gastritis among *H. pylori* seronegative participants (24.1%) compared to those who were *H. pylori* seropositive, regardless of whether they were negative or positive for CagA IgG antibody: 5.7% and 6.0%, respectively, (*p* < 0.001) [42]. Likely, participants who were *H. pylori* sero-negative, but had atrophic gastritis, represent patients with the most severe form of gastric atrophy, which resulted in the loss of *H. pylori* infection [43]. Hence, *H. pylori* might negatively affect LTL only in a subset of infected persons, those with the most severe form of gastric atrophy. These findings confirm our general hypothesis that markers of tissue damage induced by *H. pylori* are related to shorter LTL. The longer duration of *H. pylori* infection observed in older persons is congruent with the acquisition of the infection in early childhood [32, 44], and its persistence [45], unless treated. *H. pylori* infection causes gastritis and typically, with aging, the severity of gastritis increases and atrophic lesions develop in the stomach. The prevalence of atrophic gastritis increases with age [42]. Hence, our findings might also suggest that infection of longer duration might decrease LTL; nonetheless, such interpretation should be made with caution, since information on the time in which the infection was acquired is not available.

On the first impression, our results might seem to contradict our expectations. In fact, our findings fit well with the natural history of *H. pylori* infection, indicating that despite the high prevalence of *H. pylori* infection of 44% globally [46], generally *H. pylori* does not cause disease, and only some infected persons develop peptic disease and gastric cancer (reviewed in [34]). The magnitude of the difference in LTL between persons with past *H. pylori* infection and atrophic gastritis and those who are *H. pylori* sero-negative and lacking atrophic gastritis was large (unadjusted mean difference − 0.32 kb [95% CI − 0.55, − 0.10]), *p* = 0.028. After adjustment for age, sex, and religiosity, the association was slightly attenuated but remained significant (beta coefficient − 0.21 kb [95% CI − 0.41, − 0.001]), *p* = 0.049. A case-control study of gastric cancer in Poland showed an increased risk for gastric cancer in relation to short LTL [47]. The same study showed among the control group, shorter LTL in persons positive than negative for *H. pylori* [47]. A study of non-neoplastic gastric mucosa from 106 cancer-free persons linked epigenetic changes, namely, *H. pylori* related hypermethylation of the promoter CpG island, with increased severity of gastritis and the development of atrophy (as measured by the PGI:PGII ratio); while shortened telomere increased the risk for hypermethylation [48]. Collectively, these and our observations shed light on the development of damage to the gastric mucosa in relation to *H. pylori* infection, in which telomere length shortening seems to play an important role. A small cross-sectional study of 163 US adults that examined associations of sero-positivity to four persistent pathogens (CMV, HSV-1, *H. pylori,* and *Chlamydia pneumoniae*) with total pathogen burden on LTL, showed reduced LTL in relation to CMV sero-positivity and increased pathogen burden in women (*n* = 100) but not in men (*n* = 63) [30]. LTL did not differ significantly according to *H. pylori* infection and no markers of atrophic gastritis were assessed [30]. Elsewhere, among ~ 400 participants aged 53–76 years, no significant association was found between CMV IgG sero-prevalence and LTL [49], but telomerase activity was reduced in relation to CMV positivity. Taken together, these and our findings suggest that exposure to persistent infections might play a role in LTL. Our findings suggest that strong specific inflammation of the stomach, as typically induced by *H. pylori*, is negatively associated with LTL. Longitudinal studies are needed to assess the directionality of the association and possible mechanisms between infections and LTL.

Reduced LTL in relation to obesity, and longer LTL in relation to physical activity were observed only in the bivariate analysis of the current study; such associations were not significant in the multivariable models. Smoking was not associated with LTL in our sample. These observations confirm our previous report [15] that was based on a sub-sample (*n* = 250) of the current cohort. Of interest, short LTL was strongly and positively associated with the prevalence of asymptomatic coronary atherosclerosis in that analysis [15].

Unlike previous findings [46], we found a negative association between religiosity and LTL, which was attenuated and became non-statistically significant in the multiple linear regression model that adjusted for age and sex. This suggests that the association between religiosity and LTL might result from confounding. Indeed, more women than men reported being religious (Table 1), and also older than younger participants (see Additional file 3).

Our study has some limitations. Using serum PGs to study atrophic gastritis might have limited sensitivity, which might result in non-differential misclassification of atrophic gastritis. The directionality of the associations of *H. pylori* sero-status and serological evidence of atrophic gastritis with LTL remain unknown because of the cross-sectional study design. Information on previous *H. pylori* eradication therapy was not collected, given the nature of our study, which utilized archived specimens and data that were obtained in a study on cardiovascular risk factors almost one decade ago. Persons with atrophic gastritis might change their dietary habits, yet such information was not available. Therefore, we cannot rule out the possibility of residual confounders.

The response rate to participate in the original study was 77% among Arabs; we cannot exclude the possibility that the non-responders might have different characteristics than the responders.

Our study has a number of strengths including the large general population sample with the representation of both sexes and various age groups, the attainment of findings of broad generalizability, the comprehensive assessment of demographic and lifestyle factors in addition to *H. pylori* and serum PGs, and the adjustment for confounders.

## Conclusions

In this cross-sectional study, *H. pylori* IgG sero-positivity per se was not related to shorter LTL. However, persons lacking *H. pylori* IgG serum antibody with serological evidence of atrophic gastritis had shorter LTL than did those without atrophic gastritis, independent of other factors that might affect LTL.

## Methods

### Study design and population

We used archived anonymized specimens obtained in the framework of a cross-sectional study conducted during 2004-2008 among Jewish and Arab residents of Jerusalem. Details of the study design have been reported [15, 42, 50–52]. The current study was limited to the Arab participants for whom LTL measurement was performed.

The sampling frame included all permanent residents of East and West Jerusalem between ages 25–74 years, as recorded in the Israeli National Population Register. Random samples were drawn from the register for both population groups, stratified by sex and by 10-year age groups, 200 names in each stratum, for a total of 2000 names and addresses in each population. These were all invited to participate in the study. Individuals were ineligible if they were unable to provide informed consent, institutionalized, housebound, or had a severe illness; and women, if they were pregnant or gave birth within the 3 months preceding study initiation. The response rate among Arabs was 77% (*n* = 970) [15, 50]. We did not perform an a priori power calculation for the current study; we used all the available serum samples, 934/970 representing 96.3% of the Arab participants in the original study.

### Data collection and definitions of the variables

Data were collected through personal interviews with the participants conducted during 2004–2008. Information was obtained on sex and age in years. Since the data were collected during a 4-year period, we considered age at examination, grouped here as 27–34, 35–44, 45–54, 55–64, and 65–78 years).

Self-reported education was classified into three categories: as having an academic degree, completed high school, and did not complete high school [42, 50, 52]. Marital status was defined as being married or not married (being single, divorced, or widowed). The variable religiosity was assessed in view of previous reports linking between religiosity and beneficial health outcomes [53–55], and reports on associations between religiosity and LTL [46]. Religiosity was defined based on the participants’ reply to a single question: “How do you define yourself?” The possible responses were very religious, religious, traditional, and secular. Since only two participants defined themselves as very religious and only 40 participants defined themselves as secular, the responses were grouped into two categories: religious vs. traditional/secular. Self-reported number of siblings was defined as having 0–3, 4–7, or ≥ 8 siblings. Smoking was classified as reported smoking of at least one cigarette daily vs. no smoking/other (i.e., smoking less than one cigarette/day). Physical activity was evaluated using the Multi-Ethnic Study of Atherosclerosis questionnaire [50], which assesses all-domains of physical activity including leisure, transport, work, and at home. Physical activity was defined following the World Health Organization (WHO) recommendation for physical activity in adults aged 18–64 years [56]. Participants were classified as sufficiently physically active if they met the WHO recommendation of doing at least 150 min of at least moderate intensity aerobic physical activity; 75 min of at least vigorous physical activity or an equivalent combination of moderate and vigorous intensity physical activity of at least 600 metabolic equivalents (METs) minutes weekly [56]. Participants were classified as highly active if they met the physical activity level associated with health benefits (i.e., doing at least 300 min of moderate-intensity aerobic activity throughout the week; or doing at least 150 min of vigorous-intensity aerobic activity; or an equivalent combination of moderate and vigorous intensity physical activity of at least 1500 METs minutes weekly). Height and weight were measured with light clothing and without shoes. Weight in kilograms was measured to the nearest 100 g using an analog scale. Standing height was measured to the nearest 0.1 cm. BMI was calculated as weight (in kg)/height (in meters [m])^2^. Obesity was defined as BMI ≥ 30 kg/m^2^.

### Laboratory methods

Sera were tested for the presence of specific *H. pylori* IgG antibodies (Enzygnost® Anti-*Helicobacter pylori* II/IgG kit, Siemens Diagnostics Product GmbH, Marburg, Germany). The sensitivity and specificity of the kit are 94–98%. The presence of IgG antibody against recombinant CagA protein was measured in *H. pylori-*positive sera following an in-house ELISA protocol as previously described [42, 52, 57]. *H. pylori* sero-status was defined as (1) *H. pylori* negative if participants lacked *H. pylori* IgG antibody; (2) *H. pylori*-positive CagA negative, if they had *H. pylori* IgG antibody but lacked CagA IgG antibody; (3) *H. pylori*-positive CagA positive if they had both *H. pylori* and CagA IgG antibodies.

The level of serum PGI and PGII was quantified by ELISA (Biohit Inc., Helsinki, Finland) and the ratio of PGI:PGII was calculated. Serological evidence of atrophic gastritis was defined as a serum PGI level of < 30 μg/L or PGI:PGII ratio of < 3.0, as recommended by the manufacturer. Participants were also classified according to *H. pylori* IgG sero-positivity and serological evidence of atrophic gastritis as (1) *H. pylori* negative, no atrophic gastritis; (2) *H. pylori* positive, no atrophic gastritis; (3) *H. pylori* positive plus atrophic gastritis; or (4) *H. pylori* negative plus atrophic gastritis. Since atrophic gastritis is caused mainly by *H. pylori* infection and severe gastric atrophy results in loss of *H. pylori* infection [43], persons who tested negative to *H. pylori* but had atrophic gastritis were considered as having past *H. pylori* infection. All serological assays were performed in one laboratory at Tel University by an experienced technician who was masked to the LTL results and information on the other independent variables.

The measurement of LTL was performed using southern blot analysis of the terminal restriction fragment length at the laboratory of Professor Abraham Aviv, the Center of Human Development and Aging, Rutgers, The State University of New Jersey, New Jersey Medical School, Newark, USA [15, 49]. LTL (kb) was analyzed as a continuous variable expressed in kilobase pair.

### Statistical analysis

The study sample (overall and by sex) was described using means (and standard deviations (SDs)) for continuous variables, and frequency and percentages for categorical variables. Differences in mean LTL (kb) were examined using the Student’s *t* test for dichotomous variables (i.e., sex, religiosity, marital status, physical activity, obesity, smoking) and one-way analysis of variance (ANOVA) for categorical variables with more than two categories (i.e., age, education, number of siblings, and *H. pylori* sero-status/atrophic gastritis). The Bonferroni post hoc test was used for pairwise comparisons to adjust for multiple comparisons. The variable LTL followed approximately a normal distribution. The assumptions of equal variance in Student’s *t* test and homogeneity of variance in ANOVA were examined and met. These analyses were performed in stratification by sex, given the documented difference between men and women in LTL [8]. Interactions between sex and the independent variables were assessed as an initial step before running a pooled multivariable model.

Multiple linear regression models were fitted to assess the associations between *H. pylori* sero-status/atrophic gastritis and LTL while adjusting for demographic and lifestyle factors. *H. pylori* sero-status/atrophic gastritis was enforced into the model as the main independent variable of interest. Other independent variables were selected to be included in the model based on prior knowledge; if the independent variables were associated with LTL (age, sex [5, 7, 8], religiosity [46], education as a measure of SES [23], smoking [24, 25], obesity [25], and physical activity [27, 28]), or with *H. pylori* infection (education and number of siblings [58]). Categorical variables were included in the model as dummy variables. Covariates were excluded from the analysis if they were associated with LTL in the model with *p* > 0.2, and if they yielded a change of less than 10% in the point estimate [59] of the association between *H. pylori* sero-status/atrophic gastritis and LTL (suggesting that they were not confounders), or in the adjusted *R*^2^. This resulted in two multivariable models, for each of them we reported the *p* value, *F* statistics, and adjusted *R*^2^. Interactions between *H. pylori* sero-status/atrophic gastritis and the other independent variables were assessed in the models. For each independent variable, we reported the beta (slope) coefficient (and 95% CI) that were obtained from these models. The assumptions of the linear regressions were assessed and met in all models. Collinearity between the independent variables was assessed using VIF. Statistical significance was set at *p* < 0.05. Data were analyzed using IBM SPSS version 25 (Armonk, NY, USA).

## Additional files

Additional file 1: The results of the sex-stratified analysis of the mean leukocyte telomere length (kb) according to sociodemographic and lifestyle factors. (PDF 192 kb)

Additional file 2: The results from the additional multiple linear regression model of adjusted associations of demographic and lifestyle factors, *H. pylori* sero-status, and serological evidence of atrophic gastritis with leukocyte telomere length (kb). (PDF 170 kb)

Additional file 3: The self-reported responses regarding religiosity by age groups. (PDF 6 kb)

## Abbreviations

ANOVA: One-way analysis of variance
BMI: Body mass index
CagA: Cytotoxin-associated gene A
CI: Confidence intervals
CMV: Cytomegalovirus
ELISA: Enzyme-linked immunosorbent assay
*H. pylori*: *Helicobacter pylori*
HSV-1: Herpes simplex virus type 1
kb: kilo base pairs
kg: kilogram
IgG: Immunoglobulin G
LTL: Leukocyte telomere length
m: meter
METs: Metabolic equivalents
PG: Pepsinogen
SD: Standard deviation
VIF: Variance inflation factor
WHO: World Health Organization
