# Supplementary material for: Helicobacter pylori infection, serum pepsinogens as markers of atrophic gastritis, and leukocyte telomere length: a population-based study
Source: Hum Genomics. 2019 Jul 22;13:32. doi: 10.1186/s40246-019-0217-3 (PMC6647065; doi:10.1186/s40246-019-0217-3)
Supplement: Supplementary file 1 — The results of the sex-stratified analysis of the mean leukocyte telomere length (kb) according to sociodemographic and lifestyle factors. (PDF 192 kb) [file 40246_2019_217_MOESM1_ESM.pdf]

Additional file 1: Mean leukocyte telomere length (kb) according to sociodemographic and lifestyle factors, stratified by sex

	Men				Women				
	**Total**	**Mean (SD)**	**Mean difference (95% CI)**	**p value**	**Total**	**Mean (SD)**	**Mean difference (95% CI)**	**p value**	**p for interaction by sex**
**Age, years**	df=4			<0.001*	df=4			<0.001*	0.12
27-34	71	7.14 (0.55)	Reference		56	7.24 (0.54)	Reference		
35-44	97	7.06 (0.52)	-0.08 (-0.30, 0.20)		94	6.94 (0.56)	-0.30 (-0.56, -0.04)		
45-54	110	6.73 (0.57)	-0.41 (-0.66, -0.19)		95	6.88 (0.52)	-0.36 (-0.62, -0.10)		
55-64	100	6.57 (0.55)	-0.57 (-0.82, -0.33)		95	6.67 (0.57)	-0.57 (-0.83, 0.31)		
65-78	118	6.35 (0.56)	-0.79 (-1.03, -0.56)		98	6.47 (0.53)	-0.77 (-1.03,-0.50)		
**Education**	df=2			0.3**	df=2			0.001**	0.2
Did not complete high school	291	6.70 (0.60)	Reference		307	6.74 (0.56)	Reference		
Completed high school	118	6.77 (0.59)	0.07 (-0.10, 0.23)		92	6.92 (0.64)	0.18 (0.01, 0.35)		
Academic education	86	6.80 (0.73)	0.10 (-0.08, 0.28)		38	7.03 (0.64)	0.29 (0.05, 0.54)		
**Number of siblings**	df=2			0.3***	df=2			0.3***	0.7
0-3	45	6.66 (0.63)	Reference		42	6.68 (0.59)	Reference		
4-7	222	6.71 (0.63)	0.05 (-0.20, 0.30)		201	6.81 (0.63)	0.13 (-0.12, 0.36)		
≥8	228	6.77 (0.62)	0.11 (-0.14, 0.35)		195	6.82 (0.55)	0.14 (-0.10, 0.38)		
**Religiosity**				0.003				0.017	0.6
Religious	161	6.61 (0.61)	-0.18 (-0.3, -0.06)		189	6.73 (0.62)	-0.13 (-0.25, -0.02)		
Traditional/secular	334	6.79 (0.62)	Reference		246	6.86 (0.57)	Reference		
**Marital status** ****				0.3				<0.001	0.009
Married	467	6.72 (0.63)	-0.11 (-0.35, 0.12)		297	6.88 (0.59)	0.24 (0.11, 0.35)		
Not married	29	6.83 (0.54)	Reference		139	6.64 (0.57)	Reference		
**Smoking**				0.2				0.6	0.8
≥1 cigarettes/ day	202	6.77 (0.58)	0.07 (-0.05, 0.18)		39	6.84 (0.63)	0.05 (-0.15, 0.24)		
No smoking/other	293	6.70 (0.66)	Reference		394	6.80 (0.59)	Reference		
**Obesity**				0.10				0.018	0.6
BMI <30 kg/m^2	338	6.76 (0.63)	0.10 (-0.02, 0.20)		184	6.88 (0.58)	0.14 (0.02, 0.25)		
BMI ≥30 kg/m^2	158	6.66 (0.61)	Reference		254	6.74 (0.60)	Reference		
**Sufficient physical activity level**				0.17				<0.001	0.07
No	68	6.63 (0.65)	-0.11 (-0.27, 0.05)		112	6.58 (0.56)	-0.29 (-0.41, -0.16)		
Yes	428	6.74 (0.62)	Reference		326	6.87 (0.58)	Reference		

**High physical activity level**				0.035				<0.001	0.14
No	111	6.62 (0.63)	-0.14 (-0.27, -0.01)		154	6.63 (0.59)	-0.27 (-0.39, -0.16)		
Yes	385	6.76 (0.62)	Reference		284	6.90 (0.57)	Reference		

BMI: body mass index; CI: confidence intervals; df: degrees of freedom; kb: kilo base pairs; kg: kilogram; m: meters;

[*] ANOVA for the difference between the groups. Bonferroni test in men 27-34 vs. 35-44 (p=1.0), p<0.01 for all other pairwise comparisons. Bonferroni test in women 27-34 vs. 35-44 (p=0.013), 27-34 vs. 45-54 (p=0.001), 35-44 vs. 45-54 (p=1.0), 35-44 vs. 55-64 (p=0.007), 45-54 vs. 55-64 (p=0.10), 65-78 vs. 55-64 (p=0.095), p<0.001 for all other pairwise comparisons.

[**] ANOVA for the difference between the groups. Bonferroni test in men: p>0.5 for all pairwise comparisons. Bonferroni test in women: Did not complete high school vs. completed high school (p=0.028). Did not complete high school vs. academic education (p=0.011) Completed high school vs. academic education (p=0.9).

[***] ANOVA for the differences between the groups. Bonferroni test p>0.8 and p>0.4 for all pairwise comparisons in men and women, respectively.

[****] Not married included persons who reported being single, widowed or divorced
