# Supplementary material for: Helicobacter pylori infection, serum pepsinogens as markers of atrophic gastritis, and leukocyte telomere length: a population-based study
Source: Hum Genomics. 2019 Jul 22;13:32. doi: 10.1186/s40246-019-0217-3 (PMC6647065; doi:10.1186/s40246-019-0217-3)
Supplement: Supplementary file 2 — The results from the additional multiple linear regression model of adjusted associations of demographic and lifestyle factors, H. pylori sero-status, and serological evidence of atrophic gastritis with leukocyte telomere length (kb). (PDF 170 kb) [file 40246_2019_217_MOESM2_ESM.pdf]

Additional file 2: Multiple linear regression model of adjusted associations of demographic and lifestyle factors, *H. pylori* sero-status and serological evidence of atrophic gastritis, with leukocyte telomere length (kb)

	Beta coefficient (95% CI)	p value
**Sex**		
Males	-0.10 (-0.18, -0.01)	0.02
Females	Reference	
**Age, years**		
25-34	Reference	
35-44	-0.18 (-0.30, -0.05)	0.006
45-54	-0.38 (-0.51, -0.26)	<0.001
55-64	-0.56 (-0.68, -0.43)	<0.001
65-78	-0.75 (-0.88, -0.62)	<0.001
**Religiosity**		
Religious	-0.06 (-0.14, 0.02)	0.12
Traditional/secular	Reference	
H. pylori **sero-status/atrophic gastritis** *		
H. pylori negative, no atrophic gastritis	Reference	
H. pylori positive, no atrophic gastritis	0.07 (-0.05, 0.18)	0.2
H. pylori positive plus atrophic gastritis	0.18 (-0.02, 0.37)	0.08
Past *H. pylori* infection (IgG sero-negative) plus atrophic gastritis	-0.19 (-0.40, 0.01)	0.06
**Marital status**		
Married	Reference	
Not married	-0.01 (-0.11, 0.10)	0.8
**Education**		
Did not complete high school	Reference	
Completed high school	-0.01 (-0.10, 0.08)	0.7
Academic education	0.06 (-0.05, 0.18)	0.2
**Smoking**		
Regular smoking ≥1 cigarette/ day	0.02 (-0.07, 0.11)	0.7
Other	Reference	
**Obesity**		
BMI <30 kg/m^2	Reference	
BMI ≥30 kg/m^2	-0.02 (-0.09, 0.06)	0.7
**High physical activity level**		
No	Reference	
Yes	0.03 (-0.06, 0.11)	0.5
**Number of siblings**		
0-3	Reference	
4-7	-0.03 (-0.16, 0.10)	0.6
≥8	-0.05 (-0.19, 0.08)	0.4

BMI: body mass index; CI: confidence intervals; IgG: immunoglobulin; kb: kilo base pairs; kg: kilogram; m: meters;

*Atrophic gastritis was defined as serum pepsinogen (PG) I<30 µg/L or PGI: PGII<3.0.

Model summary: adjusted R^2 = 0.193, degrees of freedom=17, (F statistic =13.86), p<0.001.

Adjusted for the variables in the model.
