# Supplementary material for: Helicobacter pylori infection, serum pepsinogens as markers of atrophic gastritis, and leukocyte telomere length: a population-based study
Source: Hum Genomics. 2019 Jul 22;13:32. doi: 10.1186/s40246-019-0217-3 (PMC6647065; doi:10.1186/s40246-019-0217-3)
Supplement: Supplementary file 3 — The self-reported responses regarding religiosity by age groups. (PDF 6 kb) [file 40246_2019_217_MOESM3_ESM.pdf]

Additional file 3: Self-reported religiosity by age group

Age groups, years	Total	Self-reported religiosity, n (%)
27-34	123	39 (31.0%)
35-44	189	56 (29.6%)
45-54	205	62 (30.2%)
55-64	195	84 (43.1%)
65-78	215	109 (50.7%)
